# The role of personal and psychological, social and environmental factors in determining physical activity in Turkish school-aged adolescents: in the context of a socio-ecological model

**DOI:** 10.3389/fpsyg.2026.1822323

**Published:** 2026-05-21

**Authors:** Ferhat Çifçi, Abdurrahman Demir

**Affiliations:** 1 School of Physical Education and Sports, Dicle University, Diyarbakır, Türkiye; 2 School of Physical Education and Sports, Siirt University, Siirt, Türkiye

**Keywords:** adolescent, motivation, physical activity, physical environment, social support

## Abstract

**Purpose:**

This study aimed to examine the role of gender, grade level, academic achievement, socioeconomic status, body mass index, physical environment, motivation, and social support in predicting physical activity among Turkish adolescents of secondary school age.

**Methods:**

A correlational survey model was employed in the study. The sample consisted of 2,866 middle school students in Türkiye, including 1,598 girls and 1,298 boys. Data were collected using the Physical Activity Questionnaire for Children, the Self-Motivation Inventory, the Social Support Scale, and a Personal Information Form. Multiple regression analysis was used to analyze the data.

**Results:**

Regression analysis demonstrated that gender (*β* = 0.061, *p* < 0.001), grade level (*β* = −0.209, *p* < 0.001), academic achievement (*β* = −0.091, *p* < 0.001), physical environment (*β* = 0.086, *p* < 0.001), BMI (*β* = −0.087, *p* < 0.001), motivation (*β* = 0.251, *p* < 0.001), parental social support (*β* = 0.164, *p* < 0.001), and peer social support (*β* = 0.261, *p* < 0.001) were significant predictors of physical activity (*R* = 0.651; *R*^2^ = 0.423; *F* = 232.976; *p* < 0.001). It was also determined that these variables accounted for 42.3% of the variance in physical activity, and that peer social support exhibited a stronger standardized relationship with physical activity compared to the other variables.

**Conclusion:**

The findings indicate that Turkish middle school students engage in physical activity at a moderate level. Gender, grade level, academic achievement, physical environment, BMI, motivation, and both parental and peer support were found to be significant factors influencing physical activity participation. The study also suggests that additional factors may affect physical activity behavior among Turkish adolescents attending middle school.

## 1 Introduction

Approximately 75% of global mortality is attributable to non-communicable diseases (NCDs)—including cardiovascular disorders, malignancies, chronic respiratory conditions, and diabetes—predominantly arising from the complex interplay of genetic, physiological, environmental, and behavioral determinants (World Health Organization, 2023). Among the modifiable risk factors, physical inactivity has been identified as a major contributor to the global burden of disease (Varela et al., 2018). Data from the Institute for Health Metrics and Evaluation (2023) estimates that insufficient physical activity is responsible for approximately 830,000 deaths annually. Accordingly, World Health Organization (2009) has classified physical inactivity as a critical public health issue. Conversely, regular physical activity engagement has been shown to prevent and mitigate the progression and severity of these high-mortality conditions (Pedersen and Saltin, 2015). Furthermore, physical activity is a vital determinant of holistic well-being, encompassing psychological, emotional, and social dimensions, enhancing overall quality of life (World Health Organization, 2024).

Despite the indisputable benefits of regular and adequate physical activity across all age groups, numerous studies have reported that physical activity levels are generally insufficient and tend to decline with increasing age, most notably during adolescence (Hollis et al., 2017; Sallis et al., 2000). For instance, data from the U.S. National Health and Nutrition Examination Survey indicate that while 41.9% of children aged 6–11 meet the recommended levels of physical activity, this proportion drops to 15.3% among adolescents aged 12–17 (Centers for Disease Control and Prevention, 2022). In a global study, Guthold et al. (2020) found that only approximately 19% of adolescents aged 11–17 years met the recommended physical activity guidelines. Recognizing this global concern, World Health Organization (2024) has set a target of reducing the prevalence of physical inactivity by 15% by 2030 as part of its global action plan. WHO also encourages all countries to develop national guidelines and establish specific physical activity targets.

Accordingly, many countries, including Türkiye, have formulated and implemented national action plans aimed at promoting physical activity. Within Türkiye, the K–12 physical education, sports, play, and physical activity curricula have increasingly emphasized enhancing physical activity levels among children and adolescents and fostering lifelong habits of active living. Nevertheless, similar to global trends, significant challenges remain in effectively reducing physical inactivity. Moreover, recent data reveal that there has been little to no global improvement in physical activity participation over the past two decades and highlight persistent gender disparities (Bull et al., 2020; Guthold et al., 2020). National data further indicate consistent inequalities in participation based on age, gender, disability status, pregnancy, socioeconomic background, and geographic location. These disparities underscore the urgent need to intensify investments in physical activity promotion. Therefore, identifying the factors or antecedents associated with physical activity in this age group is critical for informing targeted strategies aimed at reducing physical inactivity and enhancing active lifestyles.

In this context, the development of targeted strategies to increase physical activity requires a multidimensional perspective that goes beyond individual-level variables. At this point, the socio-ecological model, which comprehensively addresses personal (e.g., sociodemographic, biological, psychological), social, cultural, environmental, and political factors in understanding individuals’ health behaviors and physical activity behavior, provides an important theoretical framework for understanding these behaviors and for developing effective interventions (Sallis et al., 2012). Martins et al. (2015) emphasized that psychological factors such as attitudes, perceptions, and motivation toward physical activity, alongside personal factors such as body image perceptions, social factors such as peer and family support, and environmental factors such as access to physical activity, have a decisive influence on adolescents’ participation in physical activity. Similarly, several scholars (Pan et al., 2009; Romaguera et al., 2011) have indicated that a physically active lifestyle is shaped by a combination of personal, social, and environmental determinants. Empirical studies have commonly identified gender, age, socioeconomic status, and the physical environment as major predictors of physical activity (Rossi et al., 2021). Hanifah et al. (2023) further identified screen time, access to safe and usable spaces, and cultural and social norms that prioritize academic achievement as additional determinants. Laird et al. (2016) reported that parental and peer support can influence adolescents’ physical activity engagement. In contrast to previous findings, Van Der Horst et al. (2007) found significant associations between self-efficacy, motivation, the availability of physical education and school sports, parental education, and levels of physical activity. Liu et al. (2015), in their study examining the influence of different physical activity contexts (e.g., school, gym, family), found that physical activity, particularly during adolescence, is closely linked to self-perception and self-esteem. They also concluded that school-based physical activity interventions tend to be more effective in promoting youth engagement.

While the decisive role of socio-ecological factors—such as individual, social and environmental factors—on physical activity has been established in the literature, attention is also drawn to how these factors interact to exert their influence across different samples and contexts. In this regard, numerous studies have been conducted across different cultures to identify the predictors of physical activity; however, the findings have shown considerable variation (Zaqout et al., 2016). Despite this growing body of international research, there is a lack of comprehensive studies in Türkiye that examine the determinants of physical activity among school-aged children in the aftermath of the COVID-19 pandemic. Identifying the predictors of physical activity during early adolescence—a period characterized by a marked decline in physical activity levels—could significantly contribute to the design of early intervention and prevention programs aimed at promoting long-term health outcomes. Therefore, comprehensive studies encompassing personal and psychological, social, and environmental factors within a socio-ecological model are needed to increase the physical activity levels of Turkish children and adolescents. Within this context, the aim of this study is to determine the role of gender, class level, academic achievement, socio-economic level, body mass index (BMI), physical environment, motivation and social support in determining physical activity among Turkish adolescents of secondary school age.

## 2 Methods

### 2.1 Research design

This study, conducted to identify the determinants of physical activity among Turkish adolescents of secondary school age, employed a correlational survey model. The correlational survey design is a research method used to examine whether there is a relationship between two or more variables under existing conditions, and if so, to determine the strength and direction of that relationship (Karasar, 2015).

### 2.2 Participants

Data were collected from a total of 3,114 middle school students enrolled in schools located in the cities of Mersin and Şanlıurfa. However, after excluding incomplete or improperly filled responses, the data of 2,866 students (1,598 girls and 1,298 boys) were included in the final analysis. *A priori* power analysis was conducted using G*Power to determine the sample size prior to the study. The results indicated that a sample size of at least 1,178 participants was required to detect a small effect size (*f*^2^ = 0.02) with 95% power at the *α* = 0.05 level. Participants were between the ages of 10 and 15, with a mean age of 11.76 (SD = 1.51). The participants were recruited through convenience sampling voluntarily. Convenience sampling is a non-probability sampling method in which individuals are selected based on specific criteria such as accessibility, geographical proximity, availability at a given time, or willingness to participate, often to reduce costs and expedite data collection. Within this scope, secondary school pupils in Years 5–8 who volunteered to take part in the study and for whom parental consent was obtained were included. Care was taken to ensure that participants had no physical or medical conditions preventing them from participating in physical activity, and those with such conditions were excluded from the study. Furthermore, as data was collected via self-report, students with cognitive impairments such as learning difficulties or intellectual disabilities who were receiving inclusive education were not included in the study.

### 2.3 Data collection instruments

In the present study, data were collected using the Physical Activity Questionnaire for Children, the Self-Motivation Scale, the Social Support Scale, and a Demographic Information Form developed by the researchers.

#### 2.3.1 Demographic Information Form

Participants’ demographic characteristics were obtained through an eight-item questionnaire prepared by the researchers. This form included items related to participants’ gender, age, grade level, height, weight, monthly household income, physical environment, and academic achievement. Although a variety of indicators have been utilized in the literature to represent socioeconomic status, parental average monthly income was employed as the primary indicator in the current study. In addition, students’ academic achievement was assessed based on their grade point average (GPA) from the previous academic year.

#### 2.3.2 Self-Motivation Inventory

To assess participants’ self-motivation levels, the Self-Motivation Inventory, originally developed by Dishman et al. (1980) for adults, and later adapted for children aged 10–16 by Yılmaz and Koruç (2006), was employed in this study. The inventory consists of 20 unidimensional items and is structured on a 5-point Likert scale. Higher scores indicate stronger motivation toward exercise adherence and continuity. The internal consistency coefficient (Cronbach’s alpha) reported by Yılmaz and Koruç (2006) was 0.91, while the reliability coefficient calculated for the current study was 0.78.

#### 2.3.3 Social Support for Physical Activity Scale (SSPAS)

To assess perceived social support from peers and parents in engaging in physical activity, the Social Support for Physical Activity Scale developed by Farias Júnior et al. (2014) and adapted for Turkish middle and high school students by Küçükibiş and Eskiler (2019) was utilized. The scale comprises 10 items grouped under two subdimensions: peer support and parental support. It is structured as a 4-point Likert-type scale, scored from 0 to 3. Higher scores in each subdimension reflect greater perceived social support. The internal consistency coefficients (Cronbach’s alpha) reported by the scale developers were 0.77 for the subdimensions and 0.81 for the total scale (Küçükibiş and Eskiler, 2019). In the present study, Cronbach’s alpha values were 0.85 for parental support, 0.81 for peer support, and 0.88 for the total scale. Upon examining the convergent validity of the scale, it was found that the average variance explained (AVE) values for both factors were above 0.50 (0.623 for parental support and 0.606 for peer support). Furthermore, the composite reliability (CR) values being above 0.70 (0.89 for parental support and 0.88 for peer support) indicate that the measurement model has high internal consistency. When these findings are considered together, it can be concluded that the scale demonstrates convergent validity.

#### 2.3.4 Physical Activity Questionnaire for Older Children (PAQ-C)

To determine the level of physical activity among participants, the Physical Activity Questionnaire for Older Children (PAQ-C), originally developed by Kowalski et al., and adapted to Turkish children aged 8–14 by Erdim et al. (2019), was employed. The questionnaire includes nine items designed to assess moderate to vigorous physical activity over the previous 7 days. Responses are rated on a 5-point Likert scale, with scores ranging from 1 to 5, where higher scores indicate a higher level of physical activity. The Cronbach’s alpha coefficient for the original adaptation was reported as 0.77 (Erdim et al., 2019). In the present study, the cronbach alpha reliability coefficient was calculated as 0.84.

#### 2.3.5 Body mass index (BMI)

In the study, BMI was used to assess the general health status of the participants using their weight and height. BMI is calculated by dividing body weight in kilograms by the square of height in meters: $\mathrm{BMI}=\mathrm{body weight}\;\left(\mathrm{kg}\right)/{\left[\mathrm{height}\;\left(m\right)\right]}^{2}$ (Meredith and Welk, 2005). Height and weight data were obtained via self-report from the participants.

#### 2.3.6 Academic achievement

Participants’ academic achievement was evaluated based on their end-of-year grade point average (GPA) from the previous academic year. For example, for a student currently enrolled in the 6th grade, the GPA from the 5th grade was considered. In Türkiye, end-of-year GPA is calculated by dividing the total weighted scores of the courses taken during the academic year by the total number of weekly course hours (Ministry of National Education, 2014). These GPA scores are recorded in the official e-School system and range from 0 to 100. In this study, GPA data were obtained through self-report by the participants.

### 2.4 Procedure

The study first obtained approval from the Ethics Committee at Artvin Çoruh University to ensure its ethical compliance. As the study was conducted with students enrolled in institutions affiliated with the Ministry of National Education, research permits were obtained from the Provincial Directorates of National Education in the cities of Mersin and Şanlıurfa, Turkey. Subsequently, data collection tools—including a personal information form, a self-motivation scale, a social support scale for physical activities, and a child physical activity questionnaire—were created using Google Forms. A guidance document was also prepared within this form, containing explanations regarding the study’s purpose, scope, and key considerations for completing the data collection tools, aimed at both parents and students. Participants were clearly informed that no personally identifiable information would be collected, and full anonymity was ensured throughout the research process. As participants were under the age of 18, a tick box was included to confirm that parental consent had been obtained to ensure the appropriateness of their participation in the study. Taking into account that data would be collected via Google Forms and that the form link would be shared with participants via the WhatsApp application, this consent process has been integrated into the online data collection system. During the data collection phase of the study, contact was made with school administrators and teachers at secondary schools in the provinces of Mersin and Şanlıurfa, and the link to the data collection tools was shared via class-parent WhatsApp groups. Students whose parents provided consent and who voluntarily wished to participate in the study completed the data collection tools to take part in the study.

### 2.5 Data analysis

Descriptive statistics such as frequencies and percentages were initially examined. The data were then analyzed using multiple linear regression analysis. Before conducting the main analyses, the assumptions of normality, linearity, and multicollinearity were assessed. In the study, the normality of the data was assessed using skewness and kurtosis, and it was determined that the skewness and kurtosis values ranged from 0.127 to −0.836. Tabachnick et al. (2013) stated that a normal distribution is present when the values of the normal distribution fall between −1 and +1. This indicates that the skewness and kurtosis values fall within the normal distribution range. Furthermore, The assumption of multicollinearity was assessed using the Variance Inflation Factor (VIF) and tolerance statistics. According to the analysis results, it was determined that the VIF values for the variables ranged from 1.040 to 1.879, while the tolerance values ranged from 0.532 to 0.962. The fact that these values fall within the thresholds recommended in the literature (VIF < 3; tolerance > 0.20) indicates that there is no issue of multicollinearity in the model. The linearity assumption was assessed by examining the scatter plot, which showed that a linear relationship exists between the variables and that the assumption is met. Additionally, outliers were identified using Mahalanobis distance, and cases determined to be outliers were excluded from the analyses. All statistical analyses were conducted using SPSS version 23.

## 3 Results

### 3.1 Descriptive statistics

As shown in Table 1, among the participants, 1,568 (54.7%) were female and 1,298 (45.3%) were male. Regarding grade level, 769 (26.8%) were in 5th grade, 822 (28.7%) in 6th grade, 625 (21.8%) in 7th grade, and 650 (22.7%) in 8th grade. The mean academic achievement (GPA) was *M* = 85.65, with a standard deviation of SD = 10.68. The monthly household income ranged from ₺2,200 to ₺23,000, with a mean income of ₺8,353 (SD = ₺4412.82). In terms of the physical environment, 653 participants (22.8%) reported it as inadequate, 1,000 (34.9%) as partially adequate, and 1,213 (42.3%) as adequate for engaging in physical activity. Participants’ Body Mass Index (BMI) ranged from 10.77 to 33.33, with an average of *M* = 19.09 (SD = 3.62). The mean score for motivation was *M* = 67.46 (SD = 10.10), while the total social support mean score was *M* = 13.67 (SD = 7.06). Subscale scores for parental support and peer support were *M* = 7.04 (SD = 3.96) and *M* = 6.63 (SD = 3.85), respectively. Lastly, the mean score for physical activity was *M* = 2.87 (SD = 0.83), indicating a moderate level of physical activity among participants.

**Table 1:** Descriptive statistics of all variables.

Variables	*n* (%)	Mean (Min–Max)	SD
Gender		1.45 (1–2)	0.50
Female	1,568 (54.7%)		
Male	1,298 (45.3%)		
Grade level		2.40 (1–4)	1.10
5th Grade	769 (26.8%)		
6th Grade	822 (28.7%)		
7th Grade	625 (21.8%)		
8th Grade	650 (22.7%)		
Academic achievement (GPA)	2,866 (100%)	85.65 (47–100)	10.68
Monthly household income (₺)	2,866 (100%)	8,353(2200–23,000)	4412.82
Physical environment for activity		2.20 (1–3)	7.83
Inadequate	653 (22.8%)		
Partially adequate	1,000 (34.9%)		
Adequate	1,213 (42.3%)		
Body mass index (BMI)	2,866 (100%)	19.09(10.77–33.33)	3.62
Motivation	2,866 (100%)	67.46 (20–100)	10.10
Social support for physical activity		13.67 (0–30)	7.06
Parental support		7.04 (3–15)	3.96
Peer support		6.63 (3–15)	3.85
Physical activity	2,866 (100%)	2.87 (1–5)	0.83

### 3.2 Correlation analysis

In Table 2, the results of the correlation analysis between physical activity and gender, grade level, household monthly income, academic achievement, physical environment, body mass index, motivation, parental social support, and peer social support are presented. The findings indicate that physical activity is positively but weakly correlated with gender (*r* = 0.105, *p* < 0.001), household monthly income (*r* = 0.058, *p* < 0.001), academic achievement (*r* = 0.096, *p* < 0.001), and physical environment (*r* = 0.260, *p* < 0.001). Additionally, moderate positive correlations were found between physical activity and motivation (*r* = 0.421, *p* < 0.001), parental social support (*r* = 0.480, *p* < 0.001), and peer social support (*r* = 0.511, *p* < 0.001). A moderate negative correlation was observed between physical activity and grade level (*r* = −0.330, *p* < 0.001), and a weak negative correlation was found with body mass index (*r* = −0.193, *p* < 0.001). The findings indicate that physical activity is moderately associated with interpersonal and personal factors such as peer support, parental support, motivation and grade level, thereby demonstrating that social support mechanisms and motivational structures play a decisive role in adolescents’ participation in physical activity. Furthermore, it is observed that participation levels in physical activity tend to decrease with grade level. Conversely, the fact that relationships with gender, academic achievement, income, body mass index and the physical environment remain at a low level suggests that the influence of these variables on physical activity is more limited.

**Table 2:** Correlation analysis results between physical activity and gender, grade level, household monthly income, academic achievement, physical environment, body mass index, motivation, parental social support, and peer social support.

Variables	*r*	*p*
Gender	0.105	0.000
Grade level	−0.330	0.000
Monthly household income	0.058	0.001
Academic achievement	0.096	0.000
Physical environment	0.260	0.000
Body mass index	−0.193	0.000
Motivation	0.422	0.000
Parental social support	0.480	0.000
Peer social support	0.511	0.000

### 3.3 Regression analysis

Table 3 presents the results of the multiple regression analysis conducted to examine the role of gender, grade level, household monthly income, academic achievement, physical environment, body mass index, motivation, parental social support, and peer social support in predicting participants’ physical activity levels. The findings revealed that gender (*β* = 0.061; *t* = 4.225; *p* < 0.001), grade level (*β* = −0.209; *t* = −13.756; *p* < 0.001), academic achievement (*β* = −0.091; *t* = −5.855; *p* < 0.001), physical environment (*β* = 0.086; *t* = 5.648; *p* < 0.001), body mass index (*β* = −0.087; *t* = −5.918; *p* < 0.001), motivation (*β* = 0.251; *t* = 16.069; *p* < 0.001), parental social support (*β* = 0.164; *t* = 8.401; *p* < 0.001), and peer social support (*β* = 0.261; *t* = 13.788; *p* < 0.001) were significant predictors of physical activity levels (*R* = 0.651; *R*^2^ = 0.423; *F*(9, 2,856) = 232.976; *p* < 0.001). It was also determined that these variables explained 42.3% of physical activity and that peer support and motivation showed the greatest standardized relationship with physical activity in the current model, respectively. However, household monthly income (*β* = 0.001; *t* = 0.070; *p* = 0.944) was not found to be a significant predictor of physical activity. The findings reveal that peer support, parental support and motivation exhibit a stronger standardized association with physical activity compared to other variables; this indicates that social support mechanisms and motivational factors play a decisive role in adolescents’ participation in physical activity. Conversely, the negative association between physical activity and academic achievement and body mass index, which increases with grade level and, in parallel, with age, suggests that an increase in these variables is associated with a decrease in physical activity levels. Furthermore, the study indicates that household monthly income, considered as a socio-economic factor, does not play a decisive role in adolescents’ participation in physical activity.

**Table 3:** Multiple regression analysis results for predicting physical activity.

Predictor variables	B	SE	*β*	*t*	*p*
Gender	0.102	0.024	0.061	4.23	<0.001
Grade level	−0.156	0.011	−0.209	−13.76	<0.001
Household monthly income	0.001	0.000	0.001	0.07	0.944
Academic achievement	−0.007	0.001	−0.091	−5.86	<0.001
Physical environment	0.091	0.016	0.086	5.59	<0.001
Body mass index (BMI)	−0.020	0.003	−0.087	−5.92	<0.001
Motivation	0.021	0.001	0.251	16.07	<0.001
Parental social support	0.034	0.004	0.164	8.40	<0.001
Peer social support	0.056	0.004	0.261	13.79	<0.001

## 4 Discussion and conclusion

In the current era, the decline in physical activity among adolescents has led to physical inactivity becoming an increasingly significant public health issue, with health effects beginning to emerge in the early stages of life (Van Der Horst et al., 2007). Therefore, many studies have focused on the factors influencing changes in physical activity. In this context, the present study examined the predictive roles of gender, grade level, household monthly income, academic achievement, physical environment, body mass index, motivation, parental support, and peer support in middle school students’ physical activity levels. The findings indicate that gender, grade level, academic achievement, physical environment, body mass index, motivation, parental support, and peer support significantly predict students’ levels of physical activity.

The findings of the current study revealed that gender is a significant determinant of physical activity, with male adolescents reporting higher levels of physical activity than their female counterparts. This result is supported by numerous studies in the literature. For example, Fan and Cao (2017), in a study involving 90,712 students aged between 9 and 17, reported that male students engaged in moderate-to-vigorous physical activity more frequently than female students. This finding has also been confirmed in studies conducted with children and adolescents in various countries (Armstrong et al., 2018). Gender differences in physical activity may be explained by socially constructed gender roles and cultural expectations (Spencer et al., 2015), as well as the physical and physiological changes associated with biological maturation (Rodrigues et al., 2010). As a result, these changes, along with gender roles and biological development, often lead to reduced physical activity among adolescent girls. Koivula (1995) also found that males are more likely to participate in physical activity to demonstrate competitiveness and skill, which may lead to increased engagement in physical activity. This suggests that physical activity serves a function in the context of social comparison, status acquisition and identity formation, particularly as peer relationships become more influential during adolescence. Furthermore, this process is closely linked to the reinforcement of socially constructed gender roles through peer groups. In contrast to the findings of this study, there are also studies in the literature that report no significant gender differences in physical activity levels (Hoos et al., 2003). This discrepancy may be attributed not only to the outdated nature of the studies reviewed by Hoos et al. (2003), but also to the fact that they were conducted in different cultural contexts using various measurement tools. Furthermore, when these differences are assessed from a socio-ecological perspective, they may arise as a result of the interaction of individual and interpersonal factors in a culture-specific manner.

The results of the study indicate that grade level is a significant predictor of physical activity and that adolescents’ participation in physical activity significantly decreases as grade level increases. This finding aligns with existing literature. For example, Kimm et al. (2022) monitored the physical activity levels of female adolescents aged 9–10 over 10 years and reported an 83% decline in their activity levels. Similarly, a study conducted in Japan found that lower-grade students were more physically active and more likely to meet physical activity guidelines compared to their upper-grade counterparts. Specifically, nearly half of the children in kindergarten and primary school met the recommended levels of physical activity, while approximately 70% of middle school students failed to do so (Ishii et al., 2015). This decline in physical activity has been observed not only in Asia and the United States but also in Europe. A study on French children reported a decrease in moderate-to-vigorous physical activity as children transitioned into adolescence (Blaes et al., 2011). Trost et al. (2002), in a study involving students from grades 1 to 12, found that both moderate-to-vigorous and vigorous physical activity were inversely related to grade level. They emphasized that the most significant differences occurred between grades 1–3 and 4–6, suggesting that the most notable age-related changes in activity occurred during the elementary years rather than adolescence. These findings point to a critical trend of declining physical activity during childhood and adolescence. Factors such as increased academic demands, reduced time allocated for structured play and sports, and shifting social and interactional priorities may account for this decline. Therefore, it is evident that intervention-based strategies should be developed and implemented to preserve and promote physical activity, particularly as student’s progress through higher grade levels.

Another variable examined in this study was whether household income level predicts physical activity in adolescents. Although the study found a weak association between family income and adolescents’ participation in physical activity, it concluded that family income is not a determinant of adolescents’ physical activity. This finding differs from the results of some studies in the literature. For instance, White and McTeer (2012) identified household income as one of the strongest predictors of children’s participation in organized sports. Similarly, Mo et al. (2005) reported that individuals from low-income families were less likely to engage in physical activity compared to their higher-income counterparts. A study conducted in schools with varying SES levels revealed that students in higher-SES schools not only participated more in physical activities but also had access to a broader range of activity options (Dagkas and Stathi, 2007). This highlights the influence of economic factors, particularly in cases where physical activities require financial investment. Costs related to sports equipment, membership fees, and transportation tend to be more accessible to adolescents from higher-SES families (Gordon-Larsen et al., 2006). Furthermore, a meta-analysis by Stalsberg and Pedersen (2010) found that adolescents with high SES were 58% more likely to be physically active. Torre et al. (2006) also reported a positive association between extracurricular physical activity and higher family SES among Italian adolescents. On the other hand, some studies have reported that adolescents from lower socioeconomic backgrounds also engage in physical activity at significant levels. For example, a study conducted with Thai adolescents found that those from low-SES families engaged in moderate-to-low intensity physical activities, although the type and intensity of these activities varied (Konharn et al., 2014). Tandon et al. (2021) also reported a positive relationship between socioeconomic status and meeting physical activity recommendations. However, among high school students, the predictive power of family affluence on physical activity participation was found to be limited. Nevertheless, adolescents from low-income families were shown to have significantly lower rates of sports participation. When these findings are considered together, it becomes clear that the effect of socio-economic status on physical activity exhibits an indirect and context-sensitive nature. Although the direct effect of family well-being varies relatively, the decline in participation among those with low incomes suggests that this relationship operates through different mechanisms. In particular, limited economic resources may indirectly influence physical activity behavior through interpersonal processes such as access to sports facilities, social support and opportunities for participation. In other words, this can be explained by the interaction of individual and interpersonal factors. In this context, it is recommended that recreational and competitive sports organizations, as well as school-based sports programs, develop strategies that acknowledge and aim to reduce such inequalities. However, unlike the findings of existing research in the literature, there is a lack of consistency regarding the direction and strength of the relationship between household income and physical activity; some studies report higher levels of physical activity in high-income groups, while others report higher levels in low-income groups. It is thought that the primary reason for these discrepancies may stem from the fact that the data were obtained via self-report from children and adolescents. Indeed, sample characteristics and data collection methods are decisive factors in research findings. Folley et al. (2018) report that physical activity data obtained via self-report are susceptible to systematic errors due to factors such as cognitive development, recall bias and social desirability bias. Winckers et al. (2015) have also reported that this situation may be more pronounced among those with lower incomes.

The findings of this study indicate that academic achievement is one of the predictors of physical activity and that there is a weak negative relationship between academic performance and physical activity. This is in line with previous studies, which have similarly shown that academic success may serve as a barrier to engaging in physical activity (Daley and Ryan, 2000; Esteban-Cornejo et al., 2014; LeBlanc et al., 2012). This phenomenon may be attributed to cultural and social norms that prioritize academic achievement over an active lifestyle (Hanifah et al., 2023). In Türkiye, for example, both secondary and higher education institutions typically admit students based on their performance in competitive entrance exams. Families place great importance on their children attending high-quality schools, often exerting pressure to ensure success in these exams. Such structural and interpersonal pressures can contribute to physical activity taking a back seat, particularly during adolescence, by leading to a reorientation of behavioral priorities. As a result, children may engage in physical activity only minimally or become physically inactive altogether. Several studies have also reported that academic pressure can act as a barrier to children’s physical activity behaviors (Martins et al., 2015; Bélanger et al., 2011). This suggests that physical activity behavior is shaped not only by individual preferences, but also by social expectations and performance-oriented norms. However, findings in the literature regarding the relationship between physical activity and academic achievement in children and adolescents are inconsistent. For instance, Kalantari and Esmaeilzadeh (2016) found no significant association between physical activity and academic performance among adolescent boys. These inconsistencies may be due to confounding variables such as socioeconomic and cultural factors (Kwak et al., 2009; Marques et al., 2018) or the reliance on self-reported measures for both academic achievement and physical activity in the current study. Furthermore, when viewed from a socio-ecological perspective, these differences can also be interpreted as a reflection of the varying interactions between individual and interpersonal factors across different contexts.

This study reveals that the physical environment plays a critical role in determining adolescents’ levels of physical activity. The findings indicate that the accessibility and design of the physical environment significantly influence adolescents’ participation in physical activity. Nevertheless, it is also necessary to investigate the mechanisms by which the physical environment influences adolescent behavior. Specifically, the presence of well-designed and accessible parks has been shown to support increased physical activity among youth (Floyd et al., 2011). This suggests that environmental conditions shape behavior not merely by providing physical access, but also through individuals’ perceived levels of safety, motivation and social interaction. Aibar et al. (2015) emphasized that environmental factors such as temperature, precipitation, and active commuting to school are among the key predictors of moderate to vigorous physical activity in adolescents. However, the effectiveness of these factors in promoting physical activity may vary. Individuals living in socioeconomically disadvantaged or physically deprived environments are reportedly more prone to negative health behaviors, such as physical inactivity (Kawachi, 2000). A meta-analysis by Ding et al. (2011) found that factors such as land-use diversity and residential density are significant determinants of adolescents’ physical activity levels. Similarly, Hanifah et al. (2023) reported that low levels of physical activity among Indonesian children were associated with a lack of safe and accessible spaces, as well as increased use of electronic devices. While access to a supportive physical environment can enhance participation in physical activity, it may not be sufficient on its own to help individuals meet recommended levels of physical activity across the population (Giles-Corti and Donovan, 2002). This indicates that the influence of the physical environment must be considered alongside other individual and interpersonal factors. Accordingly, infrastructure improvements such as the development of walking and cycling paths have been identified as important tools for promoting community-based physical activity. Rees et al. (2006) highlighted the need for safe and well-maintained facilities to enable youth to maintain an active lifestyle and stressed that such environmental enhancements play a crucial role in promoting healthier ways of living. In this context, the environmental measures in question should be viewed not merely as physical infrastructure improvements, but as part of multi-level interventions designed to support behavioral change. Increasing community-based initiatives that support physical activity, particularly projects that encourage the development of walking trails and bike lanes, may be an effective strategy for fostering healthier lifestyles.

Another important finding of this study is the role of body mass index (BMI) in determining adolescents’ levels of physical activity. It is well established that physically active children generally have lower body fat compared to their less active peers (Hills et al., 2011). Similarly, Kilincarslan (2019) reported that as children’s levels of physical activity increase, their BMI tends to decrease. A study conducted by Botelho et al. (2013) found that adolescents with lower levels of weekly physical activity had the highest BMIs. Additionally, Essiet et al. (2017) discovered that participants with normal BMI levels engaged in more physical activity than those with lower BMI levels, suggesting that individuals with normal BMI tend to be more active, which plays a critical role in maintaining a healthy weight. Research conducted among Saudi children revealed a significant negative association between physical activity levels and both general and central adiposity (Aliss et al., 2020). This finding points to the connection between sedentary behaviors, such as prolonged classroom sitting and leisure-time activities (e.g., watching TV, playing video games), and BMI. In this context, low levels of physical activity can be regarded not merely as a consequence, but also as a reciprocal process that contributes to the development of a high BMI. Similarly, Trost et al. (1999) found that children with higher BMI levels exhibited lower levels of physical activity compared to their peers. Another study by Adkins et al. (2004) reported that among girls, higher BMI levels were associated with lower physical activity participation. However, Guerra et al. (2006) found in a study of Portuguese children and adolescents that while boys’ physical activity levels were significantly related to obesity, no such association was found among girls. This situation suggests that, particularly during adolescence, perceived physical competence, body image, and social comparison processes may constrain participation in physical activity. McManus and Mellecker (2012) noted that obesity leads to cellular metabolic changes in skeletal muscle, which can impair the physical activity capacity of obese children. These findings demonstrate a strong relationship between BMI and physical activity, highlighting obesity as a significant factor influencing children’s activity levels. Accordingly, it is emphasized that interventions aimed at increasing physical activity should consider individuals’ body composition, particularly their BMI. Furthermore, these findings suggest that biological and psychological factors at the individual level, in interaction with interpersonal processes (e.g., social support and peer interactions), may shape physical activity behavior.

Motivation is another key factor in determining participation in physical activity and in maintaining a physically active lifestyle. The present study also found that motivation and social support were the variables showing the strongest standardized associations with adolescents’ participation in physical activity in the model. Research on physical activity has consistently identified motivation as a predictor of physical activity across all age groups. For instance, Jaakkola et al. (2008), in a study conducted with Finnish adolescents, reported that motivation was a determinant of physical activity. Similarly, Nogg et al. (2021) found a positive relationship between physical activity and intrinsic motivation in a study with American adolescents. Kalajas-Tilga et al. (2020) also identified a positive association between intrinsic motivation and engagement in moderate-to-vigorous physical activity among American adolescents. Chicote-López et al. (2018) found that the relationship between motivation and physical activity decreased along a continuum from intrinsic motivation to amotivation. Owen et al. (2013) reported that self-determined motivation was positively associated with both in-school and out-of-school moderate-to-vigorous physical activity among 9th-grade Austrian male adolescents. These findings are consistent with the results of the current study. However, these findings should not be considered solely within a correlational framework; it should also be specified which psychosocial processes mediate the relationship between motivation and the behavior of engaging in physical activity. The link between motivation and physical activity may be explained by adolescents’ autonomous choice to engage in physical activity and the intrinsic satisfaction they experience while doing so (Moreno-Murcia et al., 2014). When evaluated particularly within the context of self-determination theory, this suggests that meeting individuals’ needs for autonomy, competence and relatedness may increase participation in physical activity. Indeed, motivation directly influences levels of physical activity (Downs and Ashton, 2011) and supports the maintenance of purposeful behaviors such as physical activity (Hagger and Chatzisarantis, 2007). Children’s and adolescents’ participation in physical activity is often driven by intrinsic motives such as enjoyment (Pellegrini and Smith, 1998) and typically guided by motivational regulations, including self-determined benefits (Ingledew and Sullivan, 2002). In this context, it can be said that motivational processes at the individual level, in interaction with interpersonal factors such as social support, shape physical activity behavior.

Another finding of this study is that both parental and peer support are important factors in determining adolescents’ participation in physical activity. Furthermore, it was found that peer support is a stronger predictor of physical activity compared to parental support. Especially during adolescence, peer influence increasingly affects young people’s attitudes and behaviors and may even become a primary determinant. This situation can be explained by the fact that during adolescence, social belonging is sought in groups other than the parent, and the need for autonomy increases. Khan et al. (2020) emphasize that peers influence an adolescent’s self-efficacy perception more directly than parental encouragement in physical activity behavior. Parents, on the other hand, often prioritize their children’s academic activities over physical ones (Noonan et al., 2016). This parental priority enables adolescents to view physical activity as a form of leisure and a space for social interaction, and paves the way for physical activity to be shaped by peer influence. Therefore, it can be argued that peer support is more influential than parental support in promoting physical activity among adolescents. Nevertheless, adolescents in this age group still depend on their parents for access to physical activity, such as providing sports equipment, determining the type of activity, and facilitating access to activity venues (Gustafson and Rhodes, 2006). Thus, parental support remains a critical component of physical activity participation. Numerous studies in the literature have focused on social support as a determinant of physical activity in children and adolescents. The current findings are consistent with previous research, which has shown that both parental and peer support can contribute to encouraging participation in physical activity, as well as to the development and maintenance of an active lifestyle in children and adolescents (Anderson et al., 2009; Hu et al., 2021; Kahn et al., 2008; Strauss et al., 2001). In this context, the findings confirm a shift in the developmental paradigm during adolescence and highlight the need for physical activity interventions to be designed within a multidimensional framework that incorporates not only parental but also peer-focused dynamics. Furthermore, the fact that peer support emerges as a stronger predictor of physical activity compared to parental support during this period may be linked to the restructuring of behavioral regulation mechanisms, which become relatively autonomous from familial control and are reorganized around peer relationships. In this process, social approval and modeling mechanisms play a central role in determining the development of physical activity habits.

In conclusion, the present study has revealed that gender, class level, academic achievement, physical environment, BMI, motivation, parental and peer social support are determinants of physical activity. Furthermore, this indicates that, compared to other variables, peer social support and motivation have a greater influence on physical activity behavior among Turkish adolescents at secondary school level. These results indicate that, within the context of the socio-ecological model, personal and psychological, social, and environmental factors contribute to improving physical activity behavior in secondary school-aged adolescents, but psychological and social factors are most likely to support this behavior. This situation demonstrates that individual characteristics alone are insufficient to explain physical activity behavior; rather, it is necessary to consider multidimensional determinants such as motivational processes, social support and the physical environment in conjunction. Indeed, the national and international literature emphasizes that physical activity is a multidimensional behavior that must be explained through the interaction of individual factors, motivational processes, interpersonal relationships and environmental opportunities. In this context, it supports the view that interventions aimed at increasing participation in physical activity should be approached within a holistic framework that strengthens psychological processes and social support mechanisms and enhances environmental opportunities, rather than focusing solely on the individual.

### 4.1 Limitations and future research

The study provides up-to-date insights into the literature by highlighting the role of personal and psychological, social and environmental factors in determining physical activity among Turkish adolescents within the framework of the socio-ecological model; however, there are certain limitations that should be taken into account. Firstly, the study indicates that the predictor variables examined account for only 42.3% of the variance in physical activity. This suggests that there are other factors influencing Turkish adolescents’ physical activity behavior. Therefore, more comprehensive studies on physical activity could be conducted in the future by incorporating these factors. Secondly, this study was conducted using a cross-sectional design within the framework of a quantitative research approach. Studies that provide more in-depth and comprehensive data could be carried out by adopting mixed-methods research approaches, which combine quantitative and qualitative research designs. Furthermore, longitudinal studies could be conducted to examine the determinants of physical activity among Turkish children and adolescents. Thirdly, the sample is limited to two cities in Turkey, and participants were selected using convenience sampling. This may limit the generalisability of the findings to different socio-economic, cultural and geographical contexts. Furthermore, although the data has a clustered structure at hierarchical levels such as schools and cities, a single-level regression model was used in the analyses. This method indicates that the assumption of independent observations may not be fully met, raising the possibility that contextual effects at the school or city level may not have been reflected in the individual-level estimates. Therefore, this methodological limitation must be taken into account when interpreting the findings. In future studies, the use of larger samples selected via probability-based sampling methods covering different geographical regions, and the preference for multi-level analysis techniques (e.g., hierarchical linear modeling), which take into account the hierarchical nature of the data structure, will contribute to a more accurate distinction between individual and contextual effects and enhance the external validity and analytical robustness of the findings. Fourthly, the fact that certain variables in the study—such as weight, height and monthly household income—were obtained via self-report should be considered a factor that may influence the magnitude of the relationships between variables; as this situation may lead to measurement errors arising from social desirability, cognitive biases and recall bias. To mitigate these limitations, future research should utilize objective measurement tools—such as objective scales or official records—rather than self-reporting to collect such variables. Fifthly, treating categorical variables such as class level and physical environment as if they were continuous variables may constitute a methodological limitation. This could lead to differences between categories and a failure to fully satisfy the assumption of linearity, which may affect the coefficient estimates. It is recommended that these variables be analysed using dummy variables in future studies.

In addition to research conducted with these limitations in mind, it is also necessary to develop various strategies at the policy and implementation levels aimed at reducing physical inactivity and promoting an active lifestyle. In this context, policymakers, local authorities and educators have important roles to play. Policymakers should develop and implement action plans and policies that promote school-based physical activity programs designed to encourage active lifestyles among children and adolescents, both during and outside school hours. Furthermore, physical literacy—an essential component of a healthy lifestyle—should be established as one of the key learning outcomes starting from early childhood education. Given the clear impact of environmental factors, such as the physical environment, local governments should increase the availability of physical activity -friendly spaces such as playgrounds and sports facilities and ensure safe access to these areas for children and adolescents. Since parents and caregivers play a significant role in shaping physical activity behaviors, they should be educated about the importance of an active lifestyle and encouraged to participate in physical activity programs alongside their children.

## Data Availability

The original contributions presented in the study are included in the article/supplementary material, further inquiries can be directed to the corresponding author.
